# Serine Protease Autotransporters of the *Enterobacteriaceae* (SPATEs): Out and About and Chopping It Up

**DOI:** 10.3390/microorganisms7120594

**Published:** 2019-11-21

**Authors:** Pravil Pokharel, Hajer Habouria, Hicham Bessaiah, Charles M. Dozois

**Affiliations:** 1 Institut National de Recherche Scientifique (INRS)-Centre Armand-Frappier Santé Biotechnologie, Laval, QC H7V 1B7, Canada; micropravil@gmail.com (P.P.); hajer.hbr2@gmail.com (H.H.); hicham.bessaiah@iaf.inrs.ca (H.B.); 2 Centre de Recherche en Infectiologie Porcine et Avicole (CRIPA), Saint-Hyacinthe, QC J2S 2M2, Canada; 3 Institut Pasteur International Network, Laval, QC H7V 1B7, Canada

**Keywords:** serine protease autotransporters of *Enterobacteriaceae* (SPATE), autotransporters, cytotoxins, adhesins, *E. coli*, gene regulation

## Abstract

Autotransporters are secreted proteins with multiple functions produced by a variety of Gram-negative bacteria. In *Enterobacteriaceae*, a subgroup of these autotransporters are the SPATEs (serine protease autotransporters of *Enterobacteriaceae*). SPATEs play a crucial role in survival and virulence of pathogens such as *Escherichia coli* and *Shigella* spp. and contribute to intestinal and extra-intestinal infections. These high molecular weight proteases are transported to the external milieu by the type Va secretion system and function as proteases with diverse substrate specificities and biological functions including adherence and cytotoxicity. Herein, we provide an overview of SPATEs and discuss recent findings on the biological roles of these secreted proteins, including proteolysis of substrates, adherence to cells, modulation of the immune response, and virulence in host models. In closing, we highlight recent insights into the regulation of expression of SPATEs that could be exploited to understand fundamental SPATE biology.

## 1. Introduction

Bacteria have acquired a capacity to export and secrete proteins and other molecules to the cell surface in order to interact with the extracellular environment. The transport of proteins to the cell surface is achieved through a number of highly specialized protein secretion systems that release them into the extracellular milieu. In Gram-negative bacteria, which have an inner and outer membrane that contains a periplasmic space, secretion can be a two-step process involving export to the periplasmic space, and in some cases, subsequent secretion through the outer membrane. Autotransporter (AT) proteins comprise a large family with more than 1000 members that have been characterized [1]. AT proteins represent the largest family of secreted polypeptides in Gram-negative bacteria and serine protease autotransporters of *Enterobacteriaceae* (SPATEs) are a subclass of AT proteins that contain a protease domain belonging to the trypsin-like family, which typically contains a serine in the catalytic motif [1]. In recent years, considerable information has been obtained about how these proteins are assembled and secreted. Here, we review the latest findings on the AT secretion system with a recent model for transporting SPATE cargo out of the bacterial cell and in-depth updates of members of SPATEs including studies on genomic distribution, gene regulation, classification, and fate of the protein during in vitro or in vivo host interaction.

## 2. The Autotransporter Secretion Pathway

AT secretion through the outer membrane is mediated by the type V secretion system (T5SS) or AT secretion pathway. The T5SS pathway has been subdivided into five subtypes: (i) T5SS of monomeric ATs is classed as type Va secretion; (ii) two-partner secretion is classed as type Vb secretion; (iii) trimeric AT secretion is classed as type Vc secretion [2]; (iv) secretion of ATs homologous to both type Va and type Vb is described as type Vd [3]; and (v) secretion of intimins and invasins is classed as subtype Ve [4]. SPATEs are monomeric ATs that are secreted by the type Va secretion pathway.

**Figure 1:**
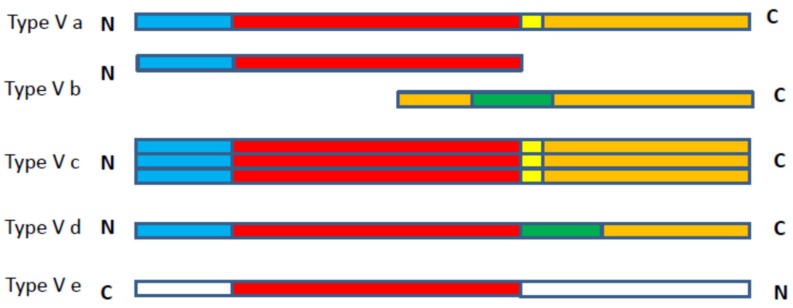
Scheme presenting domain organization among the subclasses of type-V bacterial autotransporter proteins. The labeling includes the conserved domains, colored blocks correspond to: Signal peptide (blue), passenger domain (red), polypeptide transport associated (POTRA) domain (green), linker domain (yellow) and translocation domain (orange). Adapted from [1,4].

The figure below depicts the major differences between these subtypes, which includes the variations in alignments of different domains (Figure 1). In type Va ATs, release of the N-terminal passenger domain is assisted by a C-terminal translocation domain or autoprocessed and liberated into the external milieu (explained in detail below) [1]. Type Vb is a split variant of the type Va system as the passenger domain and translocation domain are located in different polypeptide chains, and the translocated domain contains periplasmic polypeptide transport-associated (POTRA) motifs. As such, the type Vb class has also been described as a two-partner secretion system [5]. The type Vc class comprises ATs that form trimers and are also called trimeric autotransporter adhesins [2]. Type Vd ATs differ from type Va due to the presence of additional periplasmic domains between the passenger domain and the translocation domain, which is homologous to the periplasmic domains present in type Vb proteins [3]. Likewise, in type Ve ATs, the domains have a reverse order, wherein the passenger domain is at the C-terminal and translocation domain is N-terminal [4].

Understanding the biogenesis of the SPATEs is of great interest for the isolation, purification, and characterization of these proteins. Over the last two decades, a diversity of predicted AT proteins, including SPATES, have been identified through the sequencing of many bacterial genomes and through bioinformatics analysis. However, only a few SPATEs have been more extensively studied with regards to their biological functions and structural characterization. The crystal structure of the passenger domain of three SPATEs has been determined: EspP from an *Escherichia coli* O157:H7 strain [6], Hbp (also called Tsh) from an extra-intestinal pathogenic *E. coli* (ExPEC) strain [7] and Pet from enteroaggregative *E. coli* (EAEC) strains [8]. Based upon these crystal structures, general models of structure and translocation have been proposed, although, whether such models derived from only a few SPATE structures collectively represent all other SPATEs remains to be determined.

The general structure of AT proteins, including SPATES, comprises three functional domains: The signal peptide, which mediates the Sec-dependent transport of the protein into the periplasm; the N-terminal passenger domain (also called the α-domain), which is the mature protein that is exposed at the surface of the outer membrane and/or released extracellularly; and the pore forming carboxyl-terminal translocator domain (also called as β-barrel), which provides the channel through which the passenger domain is translocated to the surface of the outer membrane [9]. Initial proposals of ATs as autonomously secreted proteins have been rejected due to recent findings, indicating a role for accessory proteins located in the inner membrane [10], the periplasm [11] and the outer membrane [10] which facilitate or mediate translocation of AT proteins to the cell surface.

**Figure 2:**
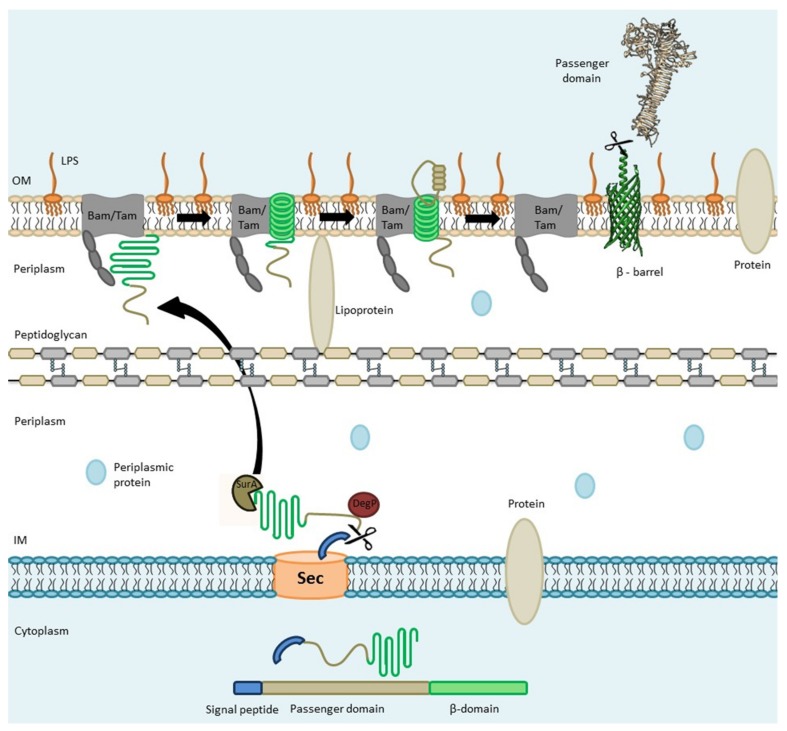
Schematic overview of Autotransporter (AT) processing, export, and secretion: The Signal peptide (SP) mediated by the Sec apparatus, guides translocation of the autotransporter to the periplasmic space. In the periplasm, the AT is kept in a “translocation competent state” by recruiting chaperones such as Skp, SurA, and DegP. Further, the Bam complex assists in the integration of the β-domain into the outer membrane and promotes the translocation of the passenger domain across the outer membrane through a hybrid-barrel mechanism wherein the AT β-barrel and Bam/Tam protein domains interact. Periplasmic chaperones such as SurA and DegP deliver an unfolded autotransporter to BamA/TamA. POTRA domains (gray in beaded structure) are contact sites for the AT protein to be transported. A hybrid-barrel is then formed by insertion of the AT β-strand through the gate region between strands 1 and 16 of the BamA/TamA barrel. Barrel expansion results in pore opening and the passenger domain can then protrude through the hybrid barrel. Subsequently, the passenger domain is released from the hybrid structure and may remain on the bacterial cell surface or can be cleaved for release from the bacterial cell. Adapted from [13,14].

ATs are exported into the periplasmic space through the Sec-dependent pathway [1], and export can occur co-translationally or follow autotransporter synthesis into the cytoplasm [12]. Immediate export following translation could improve export by preventing premature folding and degradation in the cytoplasm. Upon reaching the periplasm via the Sec-translocon and cleavage of the signal sequence, AT proteins are then protected by conserved periplasmic chaperones such as Skp, SurA, and DegP and directed toward the β-barrel assembly machinery (Bam) complex, which catalyzes the insertion and assembly of the outer membrane protein (Figure 2) [13,14]. A “Hybrid barrel-model” (Figure 2) has been proposed to explain the translocation of the passenger domain through the outer membrane. It has been shown that passenger domain secretion does not appear to use ATP, but that vectorial folding of the C-terminal of the passenger domain may contribute the necessary energy required for transmembrane passage and folding [15].

### 2.1. Sec-Dependent Export of AT Proteins through the Inner Membrane

The N-terminal signal peptide of all autotransporters mediates insertion through the inner membrane through a Sec-dependent mechanism that is common to many other exported or secreted proteins. Generally, the N-terminal signal sequences in proteins show a tripartite organization of n, h, and c regions which corresponds to N-terminal, hydrophobic, and cleavage sites [16,17]. Analysis of signal sequence mutants revealed that a hydrophobic, H region is an essential part for targeting and membrane insertion [16].

The signal peptide sequence has the function of targeting or protein translocation to the inner membrane. In *E. coli*, protein export through the Sec pathway can involve two distinct pathways: (i) The SecB/SecA pathway wherein the chaperone SecB, prevents premature aggregation or folding, keeping the protein in a “translocation—competent state” and leads to transfer to Sec A [18], (ii) the SRP (Signal Recognition Particle) pathway in which the SRP nucleoprotein complex mediates co-translational targeting by interacting with a highly hydrophobic signal sequence following translation from ribosome towards the translocon [19].

### 2.2. AT Protein Transit through the Periplasm

The mechanism of secretion from the periplasm and the transitional state of ATs while localized in the periplasm is still debated. In fact, in the periplasmic space, these proteins are prone to immature folding or aggregation and degradation by periplasmic proteases. Misfolding or degradation of ATs can be prevented apparently either by prolonged interaction with the Sec-translocon or by interaction with periplasmic chaperones [11], or both. The periplasmic localization of ATs is likely to be very transient, and translocation to the outer membrane may occur rapidly following export and processing of the signal peptide through the cytoplasmic membrane.

### 2.3. Transport of ATs through the Outer Membrane (The Hybrid-Barrel Model)

Proteins of the Omp85 superfamily such as BamA promote the insertion and folding of β-barrel outer-membrane proteins (OMPs) including AT proteins across the bacterial outer membrane [14,20]. BamA possesses five periplasmic POTRA domains which are believed to recognize substrates mediated by β-strand augmentation [21,22]. In addition to BamA, TamA has been implicated in the translocation of autotransporters [10]. TamA has structural similarities to BamA and also contains domains associated with catalytic functions like insertase and chaperone foldase activity [23]. Thus, a similar model of AT translocation has been proposed for both the BamA and TamA translocation systems.

Despite the lack of a satisfactory model for autotransporter delivery at the outer membrane, the hybrid barrel model (Figure 2) provides a plausible mechanistic model which is based on interactions with the open 16-stranded BamA and TamA barrels. The unzipped strands of these proteins can incorporate β-strands of autotransporter by β-augmentation, creating a hybrid-barrel of the AT protein with BamA/TamA. The resulting hybrid-barrel would form a pore through which the AT passenger domain would be translocated. Subsequent to passenger domain translocation through the outer membrane, a final unzipping of the hybrid complex would separate the two barrels, releasing the assembled autotransporter laterally into the outer membrane and returning BamA/ TamA to its free, uncoupled state [23,24].

### 2.4. Passenger Domain Cleavage

At the bacterial surface, the fate of AT proteins can be dependent on the specific proteins themselves as well as the physiological conditions or the environmental niche. Some ATs remain associated with the outer membrane surface whereas others, such as the Pet and EspP SPATEs, show autocatalytic activity within the β-barrel leading to cleavage of the linker and release of the passenger domain from the bacterial cell surface [25,26]. Cleavage of the passenger domain from the β-domain can take place by various mechanisms. In the case of the SPATES, the cleavage site is generally conserved.

### 2.5. A Cleavage Site is Located in the “Linker Domain” of SPATE Proteins

The linker domain encompasses an invariant 14-residue segment that spans the passenger domain and translocation domain junction in the SPATEs, and studies have shown that the cleavage site is conserved in this domain. Analysis of the (^1021^EVNNLNKRMGDL^1032^) sequence motif of EspP showed that the passenger domain is cleaved after the first asparagine residue [27,28]. The mutation in the linker peptide resulted in impaired passenger domain cleavage of EspP [28]. Similar mutations impaired passenger domain cleavage and passenger domain translocation of another SPATE, Tsh [29]. These findings suggest that the linker domain and sequence plays an important role in processing of the passenger domain. However, some SPATEs lack a twin asparagine sequence. For instance, RpeA, from a rabbit enteropathogenic *E. coli* (EPEC) strain lacks the twin asparagine residues within its linker domain and it was reported that this protein was not released into the supernatant [30]. By contrast, a recently identified SPATE called Sha (Serine protease hemagglutinin autotransporter) lacks the twin asparagine residues, but was released into the culture supernatant [31], suggesting motifs other than the twin asparagine site may be recognized for cleavage of certain SPATEs.

## 3. SPATEs

Members of the SPATE family are autotransporter proteins from a variety of enterobacterial species that all contain a consensus serine protease motif, and they have most notably been described from pathogenic *Escherichia coli* and *Shigella* spp. Although some other SPATEs have also been described in other enterobacteria including *Serratia marcescens* [32], *Salmonella bongori* [33], *Citrobacter rodentium* [34], and *Edwardsiella tarda* [35].

Other conserved architecture in SPATEs include: (1) A highly conserved secretion domain/translocation domain called a β-domain. Overall, protein homology ranges from 25% to 55% but in the case of the β-domain, homology ranges from 60% to 90% identity [36]; (2) A conserved serine protease motif (consensus GDSGSP where S is the catalytic serine) at similar positions in their N-terminal passenger domain between residues 250–270 [25,26,37,38]; (3) The serine protease motif of SPATEs does not have a role in the cleavage of the passenger domain from the β-domain [33]; (4) The passenger domain of the SPATEs are cleaved from the β-domain from a conserved cleavage site between the two asparagines [25,39]; (5) All SPATEs have unusually long signal sequences (>50 amino acids) that have been shown to facilitate post-translational targeting [40]; (6) In contrast to the classical autotransporter IgA1protease, none of the SPATEs can cleave IgA1; (7) SPATEs are highly immunogenic proteins having specific phenotypes [41].

### 3.1. Classification of SPATES

SPATEs are subdivided into class 1 and class 2 on the basis of structural and functional properties. The class 1 SPATEs are cytopathic/cytotoxic toxins (eliciting cellular changes such as cytoplasmic shrinkage, loss of membrane integrity and activation of apoptosis), likely caused by cleavage of cytoskeletal proteins such as spectrin/fodrin [41]. Further, class 1 SPATEs are believed to show cytotoxic activity primarily through targeting of intracellular substrates. On the other hand, class 2 SPATEs, the larger phylogenetic cluster, comprise O-glycoproteases that cleave mucin and other O-glycoproteins present not only on epithelial cells but also on the surface of hematopoietic cells [42]. Thus, due to mucinolytic activity, class 2 SPATEs can impart a subtle competitive advantage in mucosal colonization [41]. Sat, Pet, EspP, EspC, and SigA SPATEs belong to class 1 whereas Pic, PicU, Tsh/Hbp, Vat, EatA, and SepA belong to class 2 SPATEs.

### 3.2. Distribution of SPATEs among Intestinal and Extra-Intestinal Pathogenic *E. coli*

**Figure 3:**
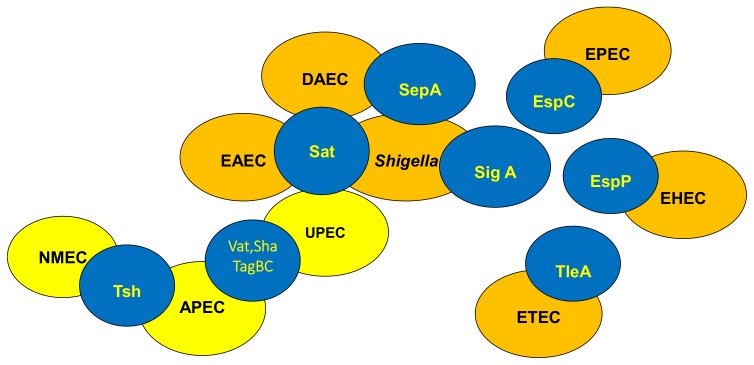
Distribution of SPATEs among intestinal and extra-intestinal pathogenic *E. coli.* SPATEs have been found not only in all recognized intestinal *E. coli* pathotypes (highlighted in orange) but also in extra-intestinal *E. coli* pathotypes (highlighted in yellow). The recent availability of many more bacterial genome sequences, has led to identification of new and previously described SPATE proteins among both human and animal pathogens, including *Salmonella*, *Citrobacter*, and *Edwarsiella*, as well as some commensal *E. coli* strains. (APEC, Avian pathogenic *E. coli*; DAEC, Diffuse Adhering *E. coli*; UPEC, Uropathogenic *E. coli*; NMEC, Neonatal meningitis *E. coli*; EAEC, Enteroaggregative *E. coli*; EHEC, Enterohemorrhagic *E. coli*; EPEC; Enteropathogenic *E. coli*, ETEC, Enterotoxigenic *E. coli*).

Some SPATEs are present in one or more pathotypes of *E coli* (Figure 3). Generally, the following SPATES are found in various groups: EspP (extracellular serine protease plasmid (pO157-encoded) from enterohemorrhagic *E. coli* (EHEC) [26], Pet (plasmid-encoded toxin) from enteroaggregative *E. coli* (EAEC) [25], Pic (protein involved in intestinal colonization) from EAEC and uropathogenic *E. coli* (UPEC) and *Shigella* [39], EspC (EPEC secreted protein C) from enteropathogenic *E. coli* (EPEC), EatA (ETEC autotransporter A) from enterotoxinogenic *E. coli* (ETEC) [43], Tsh (temperature-sensitive hemagglutinin)/ Hbp (hemoglobin protease) mainly in avian pathogenic *E. coli* (APEC) and some ExPEC (MNEC-UPEC) [37,44], Sat (secreted autotransporter toxin) from UPEC [45], and Vat (vacuolating autotransporter toxin), TagBC (Tandem autotransporter genes B and C), and Sha (Serine-protease hemagglutinin autotransporter) from APEC and UPEC strains [31,46].

### 3.3. Allelic Variation

**Figure 4:**
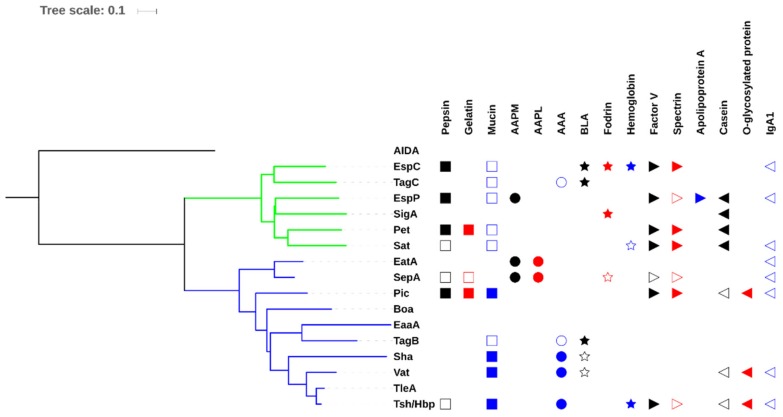
Evolutionary relationships of SPATEs based on passenger domain sequences and presentation of known protease substrates. The evolutionary history of passenger domains of characterized SPATEs was inferred using the neighbor-joining method [48]. The tree is drawn to scale, with branch lengths in the same units as those of the evolutionary distances used to infer the phylogenetic tree. The evolutionary distances were computed using the JTT matrix-based method [49] and are in the units of the number of amino acid substitutions per site. The analysis involved 17 protein sequences. All positions containing gaps and missing data were eliminated. Evolutionary analyses were conducted in MEGA6 [50]. Multiple sequence alignment was performed by Clustal W and the tree was constructed using the Mega6 software with PhyML/bootstrapping and iTOL [51]. Cluster of cytotoxic SPATEs (class 1) are in green branches while immunomodulator SPATEs (class 2) are in blue branches. SPATE protein sequences are available in NCBI database as follows: EspC, GenBank Accession No. AAC44731; EspP, NP_052685; SigA, AF200692; Pet, SJK83553; Sat, AAG30168; EatA, CAI79539, SepA, Z48219; Sha, MH899684; Pic, ALT57188; Boa, AAW66606; TagB and TagC, MH899681; EaaA, AAF63237; Vat, AY151282; TleA, KF494347; Tsh/Hbp, AF218073; AIDA, ABS20376; AAPM: MeOSuc-Ala-Ala-Pro-Met-pNA, AAPL: Suc-Ala-Ala-Pro-Leu-pNA, AAA: N-Succinyl-Ala-Ala-Ala-p-nitroanilide, BLA: N-Benzoyl-L-arginine 4-nitroanilide. The cleaved substrates, substrates that were negative for cleavage, and those untested substrates are represented by filled symbol, unfilled symbol and no symbol respectively. This comparison shows that some of the activities are phylogenetically distributed.

Phylogenetic comparisons of SPATE sequences available in the National Center for Biotechnology Information (NCBI) databases demonstrate that these proteins share high homology among their fellow members because of the high degree of amino acid identity in the C-terminal β-domains [47]. If we consider only the passenger domain, the percentage of homology drops considerably and signifies that different members contain distinct regions and have differing biological functions (Figure 4). In addition, some predicted SPATEs are related allelic variants of some of the characterized SPATEs. Despite demonstrating some closer identity to some of the characterized SPATEs, only functional testing of these proteins can confirm their specific bioactivities.

### 3.4. Confusion Due to Improper Annotation of Uncharacterized SPATE Encoding Proteins

Due to the presence of conserved domains and similarities in protein sequences among SPATEs, there are some cases where SPATE protein sequences were misnamed or given the name of a related, but distinct SPATE. For example, Vat (GenBank Accession No. AAN78874) was annotated as Tsh/Hbp in the UPEC CFT073 genome although this protein shares 78% identity with Tsh/Hbp [33,52,53]. Similarly, in the genome of *E. coli* PCN033, which was isolated from a pig with meningitis, a putative SPATE gene (GenBank Accession No. AKK51062) is named as EspC, despite this protein only having 59% identity with EspC (GenBank Accession No. WP_109867760). So, for annotations in some enterobacterial genomes, it is unfortunate that numerous uncharacterized SPATEs are incorrectly labeled as specific characterized SPATEs, despite sharing a limited degree of identity, particularly in the absence of demonstration of any biological or experimentally confirmed activities.

## 4. SPATEs Demonstrate a Diversity of Biological Activities Associated with Virulence

Since the discovery of the first SPATE, Tsh, two decades ago, research has focused on characterizing SPATEs by determining their biological activities and substrate specificities and in some cases, structural properties have been studied through crystallography. Serine protease activity is due to the GDSGS motif and this activity is inhibited by phenylmethane sulfonyl fluoride (PMSF), but not by the metalloprotease inhibitor, Ethylene diamine tetra acetic acid (EDTA) [26]. The virulence properties of SPATEs may in part be attributed to proteolytic activity. However, it is now clear that there is a staggering diversity in biological substrates and modes of action. In the next sections, we focus on specific characteristics and functions of various SPATEs (summarized in Table 1) and their possible roles in enterobacterial pathogenesis.

### 4.1. EaaA/EaaC

The EaaA and EaaC SPATEs (GenBank Accession No. Protein AAF63237.1, Nucleotide AF151091) were first identified from *Escherichia coli* reference strain ECOR-9, a human commensal intestinal isolate. These two SPATE-encoding genes were found to be associated with genes encoding non-immune immunoglobulin binding proteins that were also associated with prophages [75]. The EaaA and EaaC SPATEs are very similar 1335 aa proteins, sharing 99% identity (only 8 aa substitutions). Other than identification of these genes in ECOR-9, *eaa* gene sequences were also shown to be present in other ECOR strains (ECOR-2, ECOR-5, and ECOR-12) belonging to phylogenetic group A, but these SPATE sequences were not identified in a variety of clinical isolates [76]. However, screening of genomic sequence databases indicates EaaA/EaaC sequences are present in a diversity of *E. coli* strains (at least 50 entries of highly similar proteins identified from uniprot.org). Other than identification of the sequences in different strains, no phenotypic or biochemical properties of Eaa SPATEs have been investigated thus far.

### 4.2. EatA

EatA for ETEC autotransporter A (GenBank Accession No. CAI79539, Q84GK0; AY163491.2) is secreted by some ETEC strains and has been shown to contribute to virulence in the rabbit ileal loop model of infection, since an *eatA* mutant demonstrated less marked and slowed fluid accumulation [43]. The *eatA* sequence was identified on a plasmid, pCS1, in *E. coli* strain H10407. The EatA protein shares over 80% identity to the SepA SPATE from *Shigella flexneri*. EatA was found to degrade a bacterial adhesin EtpA, which could reduce intestinal colonization, but in parallel increased access of ETEC toxins at the host cell surface [54]. EatA was also shown to be highly immunogenic and could contribute to ETEC virulence by degrading the MUC2 protein at the small intestinal mucous layer, which could further promote access of ETEC toxins to epithelial cell surfaces [77]. The EatA protein was also shown to be present in most ETEC (over 70%) [78] and has been identified less commonly in some EAEC strains (4.1% of isolates) [79]. EatA has vaccine potential for prevention of ETEC, as it generated a high antibody response and protection against ETEC intestinal infection [77].

### 4.3. EspC

The EspC (EPEC secreted protein C) passenger domain is a 110 kDa protein (GenBank Accession No. AAC44731, U69128.1), and one of the first proteins reported to be secreted by EPEC [38]. As with other SPATEs, although EspC shares some sequence homology to IgA proteases, it cannot cleave IgA nor is the catalytic serine GDSDG motif required for release of the passenger domain from the β-domain into the external milieu [38]. It was also shown that EspC is not involved in EPEC generation of attaching and effacing (A/E) lesions, nor is it required for adherence or invasion of tissue culture cells. EspC demonstrated enterotoxic activity and increased tissue PD (potential difference) and Isc (short circuit current) of rat jejunum mounted in Ussing chambers [80]. EspC enterotoxic activity was nullified by pre-incubation with an antiserum against another SPATE, Pet [80]. Similar to Pet, EspC produced cytotoxic effects on cultured epithelial cells but with three times higher dose (120 µg/mL) than Pet. The actin cytoskeleton was disrupted, resulting in cell contraction and cell detachment. This overall effect was caused by the serine protease motif of EspC [55]. Also, similar to Pet, EspC cleaved an intracellular target, α-fodrin but the cleavage sites were different. Pet cleaves fodrin within the calmodulin binding domain between M^1198^ and V^1199^ [61]. EspC cleavage of fodrin occurred outside of the calmodulin binding domain [55]. Once inside the cells, kinetics of protein degradation indicate that purified EspC cleaves fodrin at two sites (within the 11th repetitive unit between Q^1219^ and L1^220^ and within the 9th repetitive unit between D^938^ and L^939^) which then results in disruption of focal adhesion including dephosphorylation and degradation of paxillin and FAK; leading to cell rounding and detachment [58]. However, entry to a target cell (cytosol) is critical for EspC cytotoxicity. Though internalization of purified EspC by pinocytosis was shown by [81], it cannot be considered as a natural physiological phenomenon for infection, as it took 8 h of incubation for insertion while EPEC infection delivers EspC into the cells after 30 min. Interestingly, EspC is secreted into the milieu by the T5SS and then incorporated into the T3SS translocon for entry into host cells [82]. Further, EspC has a relevant role in cell death induced by EPEC. EspC is able to induce apoptosis and necrosis in epithelial cells [56] and apoptosis could be the first event which can manifest to increased necrosis. Also, EspC was shown to interfere with the caspase cascade required for induction of apoptosis which was partially dependent on serine protease activity.

A number of biological targets recognized by EspC which are relevant to its diarrheagenic activity have been identified. Purified EspC cleaved human hemoglobin at an optimum pH between 5 and 6 [57]; although correlation of this biological property for EPEC virulence is yet to be established. EspC has also been shown to cleave other substrates like pepsin, glycoprotein coagulation factor V, and spectrin [41]. Apart from cleaving different biological substrates, EspC also cleaves bacterial components of the secretion system. EspC was found to target EspA/EspD which are translocator components of the Type III secretion system (T3SS) and control of pore formation and cytotoxicity by T3SS for the host cell [83]. The T3SS acts as a molecular syringe, comprised of pore-forming translocator proteins EspB and EspD which insert into the host cell plasma membrane and EspA which forms a hollow structure connecting the T3SS needle into the cell [84]. This result indicates that control of pore formation by EspC can support bacterial colonization, by mediating a controlled release of effector proteins from the T3SS to limit host cell death, since this could increase the immune response and potential clearance of EPEC at an early stage of infection.

### 4.4. Pet

Pet (Plasmid encoded toxin) is a 104 kDa enterotoxin produced by EAEC (GenBank Accession No. SJK83553), which has been found to increase jejunal potential difference (PD) and Isc (short circuit currents) accompanied by mucosal damage, exfoliation of cells and development of crypt abscesses [25]. Pet is encoded on the pAA plasmid and comprises a 52 aa N-terminal signal peptide and the secreted passenger domain (amino acids 53–1018), which cleaves from the β-domain between N^1018^ and N^1019^ [25].

A host-specific factor is required for proper folding of the Pet autotransporter. Interestingly, clones of *E. coli* HB101 produced both folded and misfolded variants of Pet but the wild-type EAEC only produced the properly folded active Pet, suggesting that the accessory protein from EAEC may be absent or non-functional in strain HB101 [85]. This observation shows that correct protein folding is not required for AT secretion but that accessory proteins are necessary for folding ATs in the right conformation [11,13,86]. X–ray structure of the Pet passenger domain was resolved and when compared with the most similar SPATE, EspP (50% sequence identity); Pet harbors a β-pleated sheet from residues 181–1900 whereas EspP has a coiled loop. Further, the Pet passenger domain showed more β-sheets between residues 135–143 compared to EspP [8]. These β-helices are presumed to confer functionality to the protein.

Pet produced changes in host cytoskeletal architecture in both HEp-2 and HT29 epithelial cells characterized by time and dose dependent cell elongation followed by cell rounding and detachment from the substrate, which was dependent on serine protease activity [60]. Cellular morphological changes were visible after 2 h of incubation with Pet (25 µg/mL). Pet also contributes to pathology at the mucosa which is characterized by dilation of crypt opening, extrusion of colonic enterocytes, development of intercrypt crevices and loss of apical mucus from goblet cells as a result of contraction of interlinking cytoskeleton integrity and loss of actin stress fibers and focal contacts [59]. These cytopathic effects may be due to internalization in host cells by the vesicular system as Pet activity completely vanished following incubation with brefeldin A [87]. Pet interaction with host cells requires a two-hour time lag leading to cell damage and requires a sequence of events: (1) Binding to the cell. (2) Entry into the cell by clathrin-dependent receptor-mediated endocytosis. (3) Entry into early endosomes. (4) Passage to the Golgi apparatus from endosomes. (5) Retrograde vesicular transport from the Golgi complex to the ER. (6) Delivery to the cytosol through the ER-associated degradation (ERAD) pathway [61,88]. Once internalized, loss of actin microfilaments takes place due to breakdown of cellular spectrin [60,89]. Similar to Pet, EspC also produces cytotoxic activities on epithelial cells, although the dose of EspC was three times higher and a longer incubation time was required to produce a similar result [55]. Subsequently, it was shown that the d2 subdomain of the passenger domain is required for Pet internalization by recognizing the Pet host cell receptor, cytokeratin 8 [90]; the d1 subdomain is the largest domain having a serine protease motif and was incapable of binding the cell surface without the aid of d2 subdomain [90,91]. Pet preferentially cleaves α-fodrin between M^1198^ and V^1199^ residues within the calmodulin-binding domain of fodrin’s 11th repetitive unit [61,92]. Pet-mediated cleavage of α-fodrin (spectrin) has been suggested to induce enterocyte death via apoptosis [93,94]. Together, this phenotype of Pet could explain the cellular alteration during EAEC pathogenesis [92]. Recent studies have shown that the spice, curcumin, can also affect the secretion of Pet on the bacterial surface and subsequent internalization into the epithelial cells [95].

### 4.5. Pic

Protein involved in colonization (Pic) is a 109.8 kDa extracellular protein secreted by both EAEC (GenBank Accession No. ALT57188, AF097644.1), and *Shigella flexneri* 2a [39] and also atypical EPEC [96]. Pic catalyzed mucin degradation and has also been shown to confer serum resistance and hemagglutination [39]. Further, Pic also cleaved gelatin, but did not demonstrate any activity against human immunoglobulins. In addition to Pic identified in diarrheagenic *E. coli*, another highly similar SPATE named PicU (96% amino acid identity to Pic) has been identified in some uropathogenic isolates. PicU was functionally similar to Pic from EAEC, degraded mucin and contributed to colonization during urinary tract infection (UTI) [52,62]. Interestingly, mucinase activity was not only important as a virulence factor but also may contribute to nutrient availability, since the *pic* mutant was less able to grow when compared to the wild-type strain [97]. The Pic mucinase (from all groups EAEC, UPEC, and *Shigella flexneri*) is responsible for increased secretion of mucus in the intestinal lumina of rat ileal loops by increasing mucus production in goblet cells even though this activity was independent of the serine protease motif [98]. This secretory activity of Pic favors the formation of biofilm by EAEC, a hallmark of EAEC infection. The mucolytic activity of Pic not only contributed to damage of the intestinal mucosal layer, but also cleaved mucin-type O-glycans of the immune system, including PSGL-1, CD44, CD45, CD93, and CX3CL1 [42,63]. Further, Pic significantly reduced complement activation by cleaving complement cascade factors- C3, C4 and C2 [99]. Downregulation of complement activation by Pic may contribute to EAEC, *Shigella* and UPEC infections. To add to its virulence potential, Pic was also shown to induce polymorphonuclear leucocytes/neutrophil (PMN) activation and programmed T-cell death [42].

PicU was identified in a UPEC strain and also demonstrates mucinase activity that may contribute to UTI pathogenesis [62]. To support this observation, a *picU* mutant of *E. coli* CFT073 was less able to colonize compared to wild-type parent although differences were not statistically significant. In addition, pepsin and coagulation factor V are other cleavage substrates for PicU [62]

Interestingly, a SPATE in *Citrobacter rodentium* similar to Pic and PicU, named PicC (79% identity at amino acid level), was shown to demonstrate mucinolytic activity [100]. The *picC* mutant actually outcompeted the wild-type and elicited more colitis. The PicC protease may therefore be an important immune regulator that could function to decrease stimulation of the host immune system during infection [100].

### 4.6. EspP

The extracellular serine protease plasmid (pO157-encoded) (EspP) was isolated from the culture supernatant of EHEC O157: H7 EDL933 associated with hemolytic uremic syndrome (HUS) (GenBank Accession No. NP_052685; CAA66144, X97542.1) [26] and Shiga toxin-producing *Escherichia coli* (STEC) [64,101]. PssA (protease secreted by STEC) is the homologue of EspP which differs by a single amino acid change and was found to be cytotoxic for Vero cells [102]. EspP is generally associated with STEC, EHEC and atypical EPEC [103]. In silico analysis suggests that the signal sequence of EspP is cleaved between residues A^55^ and A^56^ [26] and the 104 kDa passenger domain contains a chaperone motif and a 30 AA linker domain that connects the β-domain and the passenger domain [104]. It was later shown that biogenesis and export of EspP was not self-mediated but also required additional periplasmic chaperones SurA and DegP, which aided in the proper folding of the AT passenger domain during translocation through the Bam complex [105].

EspP proteins have been classified as four distinct alleles, namely EspPα, EspPβ, EspPγ, and EspPδ, where EspPα is associated with highly virulent EHEC O157: H7 and major non-O157 EHEC and can contribute to biofilm formation by forming macroscopic rope-like polymers which were refractory to antibiotics and showed adhesive and cytopathic effects [106]; EspPγ cleaved pepsin and human coagulation factor V, although EspPβ and EspPδ were either not secreted or proteolytically inactive [64]. Interestingly, EspPα was more prevalent in human isolates (84%) than in environmental isolates (47%) but EspPγ was more prevalent in the environment (40%) than from human sources (11%) [64,107]. The diversity of EspP alleles with different proteolytic activities necessitates specific subtyping for the screening of *espP* genes. The crystal structure of the EspP passenger domain was solved at 2.5 Å, and revealed a large β-helical stalk and a globular subdomain with the catalytic triad [6] and shared overall structure to the previously crystallized Hbp protein [7]. However, in contrast to Hbp, the active site of EspPα is slightly wider, deeper and more exposed, suggesting that it likely interacts with larger substrates [6].

Esp was shown to cleave substrates such as coagulation factor V [26], porcine pepsin A [26], apolipoprotein [65], major complement factor proteins C3/C3b but not factors H and I [66]. A valid argument for prolonged hemorrhage due to EspPα is strengthened by the ability of EspPα to cleave various serpins (serine protease inhibitors) from human plasma which are involved in blood coagulation [108]. Cleavage was specific, targeting only procoagulatory serpins such as α2-AP and α1-PI [108]. Like EspPα, EspI, another SPATE from STEC also cleaved α2-AP and α1-PI [65], but unlike EspPα, EspI mediated cleavage was not complete due to the formation of an inhibitory serpin-enzyme-complex [108]. Further, EspP was found to stimulate electrogenic ion transport in human colonoid monolayers, although this activity was Ca^2+^ dependent but independent of serine protease activity [109]. Taken together, the role of EspP in blood coagulation, pathophysiology and immunomodulation can contribute to pathogenesis of EHEC.

### 4.7. Tsh/Hbp

The first characterized SPATE, temperature-sensitive hemagglutinin (Tsh) was identified on a ColV-type plasmid in APEC strain χ7122 [37,110]. The *tsh* gene (GenBank Accession No. AF218073) encodes a protein of 1377 amino acids with a molecular weight of approximately 148-kDa. It is composed of a leader sequence that is cleaved between residues A^52^ and A^53^, a 106-kDa passenger domain that encompasses the serine protease motif (S_259_) extending from residues 53 to 1100, and a β-barrel domain of 33-kDa that extends from residues 1101 to 1377 [37]. Based on the sequence homology, Tsh was also reported in some UPEC strains [52]. However, Heimer et al. were actually screening for *vat* not *tsh*, and they erroneously named the *vat* gene in CFT073 and other UPEC as *tsh*. However, it has been reported in one study that some human ExPEC strains from newborn meningitis (11 to 50 percent) also contain *tsh* located on large plasmids similar to ColV plasmids [111]. A Tsh-like protein sharing 60% aa identity with *E. coli* Tsh was also reported in *Edwardsiella tarda*, a fish pathogen [35]. Despite the 60% identity of this SPATE from *Edwardsiella tarda* to Tsh, the bioactivity of this protein and its role in virulence fish may be quite distinct.

The production of Tsh in *E. coli* K-12 was found to be higher at low temperature (26 °C) and it conferred the capacity to agglutinate chicken erythrocytes in a mannose resistant manner. However, at higher temperatures, Tsh was released into the supernatant medium and this agglutination activity was lost leading the name, temperature-sensitive hemagglutinin [37]. Interestingly, the hemagglutination phenotype was also observed for Tsh with sheep [112], bovine, pig, turkey, rabbit, horse, and dog [31] and human erythrocytes [63]. Furthermore, Tsh promoted adherence to Caco-2 cells and to extracellular matrix proteins such as laminin, fibronectin, and collagen IV [112]. Sequence analyses indicated that Tsh is a homologue to IgA proteases (56% of similarity) of *Hemophilus influenzae* and *Neisseria gonorrhoea* [113], but was unable to cleave IgA. Tsh was shown to cleave mucin, factor V [41] and O-glycosylated proteins such as CD43, CD44, CD45, CD93, CD162, and CX3CL1 in vitro [42,63], suggesting diverse roles in cellular and immune functions. Tsh seems to also contribute directly to APEC infection, as its presence accelerates the progression of the infection and could lead to the development of lesions and deposition of fibrin in avian air sacs [37,112]. In addition to these functions, Tsh was shown to have potential enterotoxin function to induce fluid accumulation in a rabbit ligated illeal loop assay [114], although the significance of this role for Tsh in mammalian enteric disease is unknown.

Hbp (hemoglobin protease) is a near-identical variant of Tsh that was isolated from a patient with an intra-abdominal wound infection, and only differs by two amino acids (Q209K and A842T) (GenBank Accession No. AJ223631) [44]. Like *tsh*, the *hbp* gene is located on a virulence plasmid, pColV-K30. Hbp was shown to cleave hemoglobin and acquire heme in an iron depleted host niche [44]. Heme captured by Hbp could also promote growth of *Bacteroides fragilis*. *B. fragilis* was shown to contain a specific receptor that recognizes the heme-Hbp complex and it is capable of exploiting heme liberated from host complexes and cause intra-abdominal abscesses in patients [67].

### 4.8. TleA

Tsh-like ETEC autotransporter or TleA (GenBank Accession No. KF494347) is an AT that was identified in an ETEC strain [68]. It is a 4110-bp gene that encodes a 1369-amino-acid precursor. Sequence analyses have shown that TleA contains a signal peptide from residues 1 to 52, a passenger domain from residues 53 to 1092 with a serine protease motif GDSGS from residues 257 to 261 and a β-barrel domain from residues 1093 to 1369. The alignment of the passenger domain sequences of the other members of SPATEs indicated that TleA is a class 2 SPATE and shares 97% identity with the Tsh autotransporter [68]. TleA may have a role in intestinal colonization and immunomodulation as it was shown to degrade bovine submaxillary mucin and leukocyte surface glycoproteins CD45 and P-selectin glycoprotein ligand 1. Further, nonadherent *E. coli* HB101 expressing TleA conferred the capacity to adhere to Caco-2 cells, while such adherence was not observed in the wild-type ETEC strain 1766a [68].

### 4.9. Vat

Vacuolating autotransporter toxin (Vat) is a 140 kDa class II SPATE that was found to be encoded on a pathogenicity island from APEC *E. coli* Ec222, and was shown to contribute to respiratory tract infection, cellulitis and septicemia in poultry [46]. The *vat* gene is located on a pathogenicity island (VAT-PAI) between the *proA* and *yagU* genes (GenBank Accession No. AY151282). The *vat* pathogenicity island in Ec222 contains 33 ORFs wherein *vat* is ORF27. Interestingly, ORF26 share 44% aa identity with the PapX regulator from UPEC strains [46]. The *vat* predicted gene product also shares 97% identity with an AT present in UPEC strain CFT073, which was annotated mistakenly as Tsh/Hbp. However, the new annotation has been corrected as Vat-ExPEC [33,52,53]. Vat shares 77.5% identity to Tsh/Hbp of APEC [44,110], which explains why Vat has sometimes been mislabeled as Tsh for certain strains or genome sequences.

Unlike Tsh/Hbp, the secreted 111.8-kDa Vat passenger domain was unable to cleave casein-based substrate [46]. Compared to the *vat* mutant, APEC Ec222 exhibited a robust cytotoxic effect on chicken embryonic fibroblast (CEF) cells. Cytotoxic activity involved vacuole formation in cells that was visualized from 2–24 h after exposure [46]. In a cellulitis infection model, chickens challenged with *E. coli* Ec222 developed cellulitis, whereas none of the chickens developed cellulitis when infected with the *vat* mutant [46]. In UPEC, Vat elicits an antibody response in some urosepsis patients, and the titer of Vat-specific IgG was higher in the plasma of patients compared to the titer found in controls and patients infected with *vat*-negative UPEC strains [115]. Vat contributed to fitness in UPEC during murine systemic infection [116]. Further, a role for Vat in combination with other SPATEs was demonstrated for fitness in murine kidney colonization and cytotoxicity [31]. Vat has also been shown to mediate agglutination of erythrocytes and cleavage of O-glycoproteins in vitro [63].

Vat (Vat-AIEC) from an adherent invasive *E. coli* (AIEC) associated with Crohn’s disease, is 97% similar to Vat from APEC Ec222 and contains a modification in the serine catalytic domain to GDSGSP instead of the conserved ATSGSP motif present in Vat [117]. Vat-AIEC acts as a mucinase and inactivation of *vat* reduced gut colonization by one-log in a mouse model [117].

### 4.10. Sha

**Figure 5:**
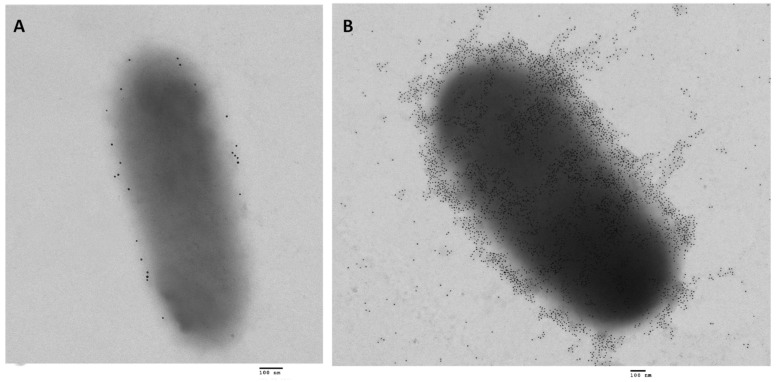
Transmission electron micrographs of *E. coli* BL21 expressing the Sha autotransporter [31] immunolabelled with 10-nm-diameter gold particles (**A**) The negative control, adsorbed serum was used for the primary incubation with *E. coli* BL21 empty vector and shows very low background of gold particles. (**B**) Gold-labelling with AT-specific antibodies shows that when Sha is constitutively expressed, proteins are localized on the surface as well as released in the supernatant. So, the protein released can gain access to host cell targets, whereas the protein associated with bacterial cells can mediate agglutination, autoaggregation, and adherence to host cells. Bar 100 nm.

A recently identified SPATE-encoding gene, *sha* (serine-protease hemaglutinin autotransporter), is located on a distinct region of a ColV-type plasmid [31]. The *sha* gene (GenBank Accession No. MH899684) was more common among APEC (present in 20% of 299 APEC strains) than in UPEC (0.9% of 697 strains). Sha is more closely related to Tsh (43% identity) and Vat proteins (38% identity) than to other SPATEs. Similar to Vat and Tsh, Sha showed elastase-like activity by cleaving N-Succinyl-Ala-Ala-Ala-p-nitroanilide. In addition, Sha increased adherence to both human and avian epithelial cells, whereas Tsh only increased adherence to bladder cells, and Vat only increased adherence to kidney cells. Sha also demonstrated hemagglutation for erythrocytes of a variety of animal species (sheep, bovine, pig, dog, chicken, turkey, rabbit, horse, and human), contributed to increased biofilm formation, and delayed cytotoxicity (release of LDH after 12 h from bladder epithelial cells). As similar carbohydrates may be present on erythrocyte surfaces, it is not surprising that Sha as well as Tsh and Vat autotransporters demonstrated extensive hemagglutination activity for a variety of erythrocytes. We believe that the contrasting phenotype when Sha is expressed in high copy vector—agglutination with erythrocytes by the protein present on the bacterial surface as well as the cytopathic effects of released protein in culture supernatant could be explained by the presence of protein both the bacterial surface and released in the supernatant (Figure 5). Although, loss of *sha* didn’t affect competitive fitness during colonization of the urinary tract of female mice, *sha* expression was upregulated six-fold in infected bladder compared to culture in lysogeny broth (LB) [31].

### 4.11. Sat

A Secreted autotransporter toxin (Sat) was identified in UPEC strain CFT073 [45] and later also described in *Shigella*, EAEC, DEAC and neonatal septicemia *E. coli* strains [118]. The 3885-bp *sat* gene (GenBank Accession No. AAG30168) is located on a pathogenicity island that also carries a *pap* fimbrial gene cluster. The *sat* gene product is a 1295-amino-acid precursor with a molecular weight of 142 kDa [45]. Sat was unable to agglutinate human erythrocytes and did not cleave glycoproteins CD43 and CD162 from Jurkat cells [63]. Sat was also shown to cleave casein, coagulation factor V and nonerythroid spectrin, but not pepsin or mucin [41,45]. Like Vat, Sat also exhibited cytotoxicity on epithelial cells, including HK-2, HEp-2 and Vero cells, characterized by vacuole formation, autophagy and cell detachment [45,69,119]. Cytotoxicity included disruption of actin and other cytoskeletal and nuclear proteins and was dependent on the serine protease active site [120]. Sat is internalized by an unknown mechanism, and shown to be localized specifically to the cytoskeletal fraction of bladder and kidney epithelial cells [120].

The autophagy in HeLa epithelial cells triggered by Sat led to disruption of the F-actin cytoskeleton [119]. Sat also modified tight junction-associated proteins ZO-1, ZO-2 and occludins in human polarized epithelial intestinal Caco-2 and TC7 cells, resulting in increased paracellular permeability [121]. In addition, Sat induced a strong immune response in a murine model of ascending urinary tract infection, although a *sat* mutant colonized urine, bladder and kidneys as well as the wild-type strain [45]. However, kidneys of mice infected with the wild-type strain showed dissolution of the glomerular membrane and vacuolation of proximal tubules and these lesions were absent in kidneys infected with the *sat* mutant [69]. In addition, Sat from a Diffusely adhering *E. coli* (DAEC) strain triggered pronounced fluid accumulation and villous necrosis in rabbit ileal tissue [122]. Taken together, Sat plays an important role as a cytotoxin in the pathogenicity of urinary tract infection as well as in intestinal infections. Still, the role of Sat as an enterotoxin is paradoxical, since Sat is also present in the probiotic strain *Escherichia coli* Nissle 1917 even though it was shown to be a functioning protease and was expressed during colonization of the mouse intestine [123].

### 4.12. SepA

*Shigella* extracellular protein A (SepA) was identified in *Shigella flexneri*, which causes diarrhea (shigellosis) in humans [70] and also in some enteroaggregative *E. coli* (EAEC) strains [103]. The *sepA* gene (GenBank Accession No. Z48219) is located on the 200 kb virulence plasmid pWR100 and encodes a 1366 a.a. precursor of 146 kDa [70]. SepA exhibited protease activity of some synthetic peptides such as Suc-Val-Pro-Phe-pNA [71], although no protease activity was found on natural substrates such as gelatin, IgA1 [70], angiotensin-I, egg lysozyme [71], fibronectin, mucin, pepsin, factor V, spectrin, or fodrin [41]. Unlike other class-2 SPATEs, SepA did not cleave O-linked glycoproteins from leucocytes such as CD43, CD44, CD45, CD93, CD162, and CX3CL1 [63]. Deletion of *sepA* did not affect entry of *S. flexneri* into epithelial cells or cell-to-cell spread; however, the *sepA* mutant showed a decrease in fluid accumulation and inflammation in the rabbit ileal loop model compared to the wild type [70]. Furthermore, in a human explant model, the *sepA* mutant demonstrated reduced mucosal damage and a significant reduction in desquamation of intestinal epithelial barrier [124]. SepA also induced disruption of the apical pole of a polarized epithelial barrier and facilitated invasion of intestinal cells by *Shigella* [125]. This was associated with a decrease in LIMK1 leading to increased accumulation of colifin, a protein involved in actin dynamics [125,126].

### 4.13. SigA

*Shigella* IgA-like protease homologue or SigA is another SPATE identified in *Shigella flexneri* 2a [72] (GenBank Accession No. AF200692). The *sigA* gene is located on the *she* pathogenicity island and encodes a 103 kDa protein [72,74]. SigA imparts cytotoxic and enterotoxic effects and was shown to cleave casein [72] and recombinant human α II spectrin (α-fodrin) [73], indicating that it might contribute to patho-physiological manifestation of *Shigella*. SigA shares 56% identity to the Pet. A *S. flexneri* 2a *sigA* mutant had 30% reduction in fluid accumulation in a rabbit ileal loop model. It was also shown that immunogenic SigA can bind to HEp-2 cells and induce cell rounding and detachment, phenotypes similar to purified Pet toxin from enteroaggregative *E. coli*, suggesting that SigA could play a key role in the virulence of *S. flexneri* [72,73]. Owing to the immunogenic properties of SigA [127], a computational approach has been used to identify potential epitopes for generation of a peptide vaccine against *Shigella* [128]. Due to their high immunogenicity, the potential of SigA and other SPATEs as vaccine targets is warranted.

### 4.14. Boa

Although most SPATEs identified to date were characterized from different pathotypes of *E. coli* and *Shigella* spp., Boa represents the only SPATE identified in *Salmonella spp*. Boa is present in *Salmonella bongori*, a Salmonella species associated mainly with reptiles, but which have been reported to infect some animals and humans. Surprisingly, no other SPATEs have been identified in all other *Salmonella enterica* serovars. It has been suggested that Boa may have been acquired by horizontal gene transfer from another Enterobacteria such as *E. coli* [74]. The *boa* gene encodes a 1384 protein (GenBank Accession No. AAW66606, FR877557) predicted to have a long signal peptide of 57 residues, a passenger domain of 1050 residues containing a serine protease GDSGS motif from residues 262 to 266 and a β-barrel domain of 277 residues [74,129]. To date, the biological properties of the Boa protein or its potential role for *Salmonella bongori* pathogenesis have not been determined.

### 4.15. TagBC

Recently, two new SPATEs referred to as “tandem autotransporter genes, *tagB* and *tagC*, located adjacent to each other on a genomic island between the conserved *E. coli* genes *yjdI* and *yjdK* in an APEC O1:K1 strain were identified [31]. Genome analysis and screening for *tagB* and *tagC* (GenBank Accession No. MH899681) indicate that these SPATE genes are present in APEC as well as UPEC strains [31]. Among 697 UPEC isolates, *tagB* sequences were present in 70 isolates (10%), whereas *tagC* sequences were present in 80 isolates. While amongst 299 APEC strains, *tagB* sequences were present in 14 isolates (4.7%) and *tagC* sequences were present in 21 isolates (7%). Interestingly, all of the *tagB* or *tagC*-positive APEC isolates were exclusively from infections in turkeys. Both proteins showed trypsin-like activity and efficiently cleaved N-Benzoyl-L-arginine 4-nitroanilide, similarly to the activity of EspC protein. Further, TagB and TagC were autoaggregating, hemagglutinating, could promote adherence to the HEK 293 renal and 5637 bladder cell lines as well as cytotoxic to human bladder cell lines when expressed in *E. coli* K-12 (with early release of LDH after 5 h). Neither of them required a functional serine protease motif for secretion [31]. In spite of these in vitro phenotypes, loss of TagBC did not have any appreciable effect on virulence or fitness of the mutant in bladder and kidneys of infected mice.

## 5. Regulation of Expression of SPATEs

Although numerous SPATEs have been characterized and their roles in infection and cell toxicity have been reported, determination of the mechanisms of regulation of SPATEs has been limited. In general, most SPATEs are thermally regulated and are better expressed under conditions similar to the host infection sites and upon contact with host cells, in tissue culture medium and at neutral to alkaline pH (7 to 9). EspP, EspC, Tsh, Pic, SigA, SepA are thermoregulated, although mechanisms of regulation remain to be elucidated [26,37,39,57,113]. Expression of the *vat-AIEC* gene was upregulated when grown at pH 7.5 with bile salts and mucin, which are similar to conditions in the distal ileal segment of the GI tract [117]. Expression of EspP in culture supernatant was higher in lysogeny broth (LB) than in minimal essential medium (MEM) as well as higher at 37 °C than at 20 °C [26,130]. Further, when EHEC 5236/96 (O26:H11) was grown in contact with human intestinal epithelial HCT-8 cells, *espP* expression was upregulated more than 35-fold [131]. Likewise, transcription of *pet* expression was increased in tryptone-containing medium, which might be of clinical significance for the milk-drinking pediatric population that can be infected with EAEC. Similarly, *tsh* and *vat* expression was shown to be upregulated in minimal medium when compared to rich LB medium [31]. Despite determining what conditions increase expression of some SPATEs, defining which regulatory mechanisms control SPATE expression has been limited. Specific aspects of regulation of expression of some SPATEs are presented below.

### 5.1. Regulation by LER

The locus of enterocyte effacement (LEE) encoded regulator (Ler) regulates the LEE pathogenicity island of EPEC and EHEC which produce attaching and effacing lesions on host intestinal epithelial cells. Ler activates the transcription of various LEE operons [132]. Apart from regulating operons associated with the LEE and its Type 3 secretion system, Ler also strongly activates the *espC* promoter (by 31-fold) and hence increases the production of the EspC SPATE in EPEC [133].

### 5.2. Regulation by H-NS

Histones are small, abundant, highly conserved proteins that have been recognized as DNA binding proteins. They play a role in compacting DNA into the nucleosome, the main structures to form chromosomes, in eukaryotic cell nuclei [134,135]. In bacteria, such as *E. coli*, some proteins have been described as histone-like proteins. They may not share the same functions as compacting prokaryotic DNA but it was shown that they play a role in the regulation of genes by competing for binding to their promoter, and these genes could be associated with virulence, osmoregulation, pH and temperature sensing [136]. Based on sequence homology, four major groups of histone-like proteins were described: histone-like proteins *Escherichia coli* U93 (HU), histone-like nucleoid structuring proteins (H-NS), integration host factors (IHF), and factors for inversion stimulation (FIS). Among these, a role for H-NS has been reported for regulation of different AT encoding genes including regulation of the SPATE Vat.

H-NS has been shown to regulate different trimeric autotransporters [137,138,139]. Further, H-NS repressed the expression of *vat*; in UPEC strain CFT073, as a Δ*hns* mutant was shown to secrete a significantly higher level of Vat than the wild type strain [115]. Further, sequence analysis of the *vat* promoter region predicted the presence of three potential H-NS binding sites in the *vat* promoter region [115]. It seems H-NS could also potentially directly regulate other SPATE genes, as there are predicted putative H-NS binding sites present in SPATE gene promoter regions (Table 2). However, further experimental evidence will be required to confirm whether or not regulation of expression of SPATEs by H-NS is a common phenomenon.

### 5.3. Regulation of Vat by the MarR-Related Protein VatX

The multiple antibiotic resistance regulator (MarR) family are proteins that regulate the expression of many genes involved in resistance to multiple antibiotics including tetracycline, chloramphenicol, β-lactams, nalidixic acid, penicillins, fluoroquinolones, toxic substances, organic solvents, oxidative stress agents and pathogenic factors [115,141,142,143,144,145]. In *E. coli*, MarR is located in the chromosome in the *mar* locus and consists of an operator marO and two divergent transcriptional units marC and marRAB [146]. Besides, the possible role of H-NS as a negative regulator of Vat, an open reading frame was located downstream of the *vat* gene designated as ORF26 in the VAT-PAI from Ec222 [46] and c0392 in CFT073 [53]. This ORF shares 44% amino acid identity to the protein PapX (P pilus-associated transcriptional regulatory) from CFT073 [115,147]. PapX has been described as a member of the MarR family, it regulates flagella by binding to the *flhDC* promoter region [115,147]. Considering identity to PapX, the ORF was named VatX. The *vatX* gene is present and adjacent to the *vat* gene in many strains. The VatX protein contains a MarR protein family (PFAM) domain (PF01047) and a helix-turn-helix motif that is characteristic of DNA binding proteins and was classified in a different clade of MarR family regulators, and is more closely related to the PapX, SfaX, and FocX fimbria-associated regulators. In UPEC strain CFT073, overexpression of VatX increased expression of Vat 3-fold compared to wild-type levels. Interestingly, in the absence of H-NS, the *vat* and *vatX* genes were both co-transcribed, suggesting VatX may compete with H-NS to promote expression of *vat* [115].

### 5.4. Co-Regulation of SPATEs by CRP and FIS Proteins

The cyclic AMP receptor protein (CRP) is a global transcription factor which is required by *E. coli* in carbon metabolism [148,149]. CRP binds as a homodimer to a 16-bp DNA binding site, is allosterically activated by cAMP binding and interacts with RNA polymerase as either an activator or a repressor of transcription initiation [150]. CRP has been identified as a key transcription factor for the *pet* gene in EAEC strain 042 [151]. In addition, transcription from the *pet* promoter was found to be co-dependent on CRP and Fis regulators and the synergy between these regulators was due to the non-optimal position for transcription initiation of CRP and the additional required binding of the Fis regulator [151]. Fis is a versatile transcription factor and is involved in site-specific recombination events, organization of local DNA topology in bacterial chromosomes, and as a global transcription factor [152,153]. It binds DNA as a homodimer that recognizes a degenerate 15-bp binding sequence often found in many promoter regions [154]. Through a co-activation mechanism, Fis was also shown to co-regulate SPATE genes with CRP including the *sat* gene from UPEC and *sigA* from *Shigella sonnei* [155].

Clearly, further investigation of regulation of SPATE expression by either DNA-binding proteins, transcriptional factors and mechanisms controlling expression of different SPATE proteins is needed. Further, potential cross-talk or hierarchical production of different SPATES needs to be considered and to what extent other virulence proteins affect the trafficking and secretion of SPATEs, if they are using the same secretion machinery such as BAM/TAM systems for their export and biogenesis at the bacterial cell surface.

## 6. Some SPATEs Can Also Mediate Degradation of Bacterial Protein Targets

The perspective of considering SPATEs uniquely as virulence proteins specifically produced to damage host cells and promote infection has been called into question in recent years. A newly described role for some SPATEs has been identified to be the degradation of bacterial proteins and secretion systems. Protease activity of SPATEs has mainly been determined for host proteins, however, importantly, some SPATEs can play an important role in infection through targeting of other bacterial substrates. The EatA SPATE from ETEC degrades the ETEC EtpA adhesin which can lead to reduced intestinal colonization [54]. Reduced colonization may be an advantage for ETEC to quickly deliver enterotoxin without causing extensive damage and may reduce inflammation and decrease the host immune response – acting as hit and run strategy of the bacteria. Similar activity was shown by EspC, wherein EspC reduced secretion of EspA and EspD, which are effector proteins of the EPEC T3SS [83]. In this case, EspC contributed to reducing pore formation and cytotoxicity by degrading EspA and EspD. A similar interchangeable activity was found for EspP [83]. Therefore, these SPATEs can also contribute to EPEC/EHEC infections by degrading other bacterial secreted proteins secreted by the T3SS before they contact the host cell. Another example of interference with other bacterial virulence factors is EspPα present in EHEC, which has been shown to degrade the pore-forming repeats-in-toxin (RTX)-protein hemolysin Ehx (EHEC- Hly) [156] in its free and vesicle-bound form [131]. EspP mediated cleavage was specific to the Ehx hydrophobic domain, which is crucial for interaction with the host cell membrane and pore formation [157]. Similarly, the reduction in the level of active pore-forming toxin may also alter the host immune response and contribute to intestinal colonization and pathogenesis.

## 7. Conclusions

Improved technologies and affordability of DNA sequencing and other methods to investigate bacterial/host interactions has greatly advanced our understanding of many aspects of bacterial pathogenesis in recent years. The large amount of data from genome sequences has also resulted in the determination of new putative SPATEs, which may also contribute to host colonization and infection. Two decades after the characterization of the first SPATE, much progress has been made in the study of SPATEs by combining in silico bioinformatics, bacteriology, molecular biology, biochemistry and cellular biology and pathogenesis studies. Within the SPATE family, all members may share a similar global structure, but each SPATE may mediate distinct functional properties and substrate specificities and be associated with certain bacterial pathotypes, the type of the disease they cause, and host animal species they infect. One would expect that the variety of host and tissues niches could result in diversification of SPATE functions. Further, these pathogens can also differ in their interactions with the host, with some SPATE-producing pathogens remaining extracellular during infection, whereas others may invade cells or survive in host phagocytes. Many questions remain to be answered about the molecular basis for substrate recognition and specificity of SPATEs and also fundamentally understanding how these proteins are regulated. It is also unclear to what extent these SPATEs may modulate or alter proteins and the bacterial surface and effect pathogenesis. Moreover, the SPATE proteins are also frequently present in certain enterobacterial pathogens commonly associated with both enteric and systemic diseases in humans and animal hosts. As these proteins are highly immunogenic, it stands to reason that the SPATEs or their conserved epitopes can be targeted for potential approaches to develop vaccines to prevent such diseases.

## Figures and Tables

**Table 1:** Summary of characteristics of different SPATEs.

SPATEs	Organism ^a^	Biological Functions	References
EatA	ETEC	Enterotoxin	[54]
EspC	EPEC	Cytotoxin, Enterotoxin Cleavage of fodrin, hemoglobin, pepsin, coagulation factor V, translocator components (EspA/EspD) of T3SSCell rounding and cell detachment	[41,55,56,57,58]
Pet	EAEC	Mucosal cytoxicity, Cleavage of spectrin, pepsin, factor V	[25,41,59,60,61]
Pic	Shigella, EAEC	Serum resistance Mucinase, Hemagglutination Colonization, Cleavage of gelatin, factor V, O-glycans: PSGL-1, CD44, CD45, CD93 and CX3CL1	[39,41,42,52,62,63]
EspP	EHEC, STEC	Cleaved pepsin, factor V, apolipoprotein, complement factors: C3/C3b and C5	[26,41,64,65,66]
Tsh/Hbp	APEC	Hemagglutinin, Binding to Caco-2 cells and to EMPs (laminin, fibronectin, and collagen IV) and heme Cleavage of mucin, factor V and O-glycosylated proteins in leukocyte	[37,41,63,67]
Sha	APEC, UPEC	Autoaggregation, hemagglutination, biofilm formation, proteolytic activity on synthetic peptide: N-Succinyl-Ala-Ala-Ala-p-nitroanilide, adherence and cytopathic effects on bladder epithelial cell line	[31]
TleA		Binding to Caco-2 cells Cleavage of bovine submaxillary mucin, leukocyte surface glycoproteins CD45 and P-selectin glycoprotein ligand 1	[68]
Vat	APEC, UPEC	Vacuolating cytotoxin, Agglutinate leukocyte Cleavage of O-glycosylated proteins in leukocyte	[46,63]
Sat	UPEC	Vacuolating cytotoxin on HK-2, HEp-2 and Vero monkey kidney cells Cleavage of casein, factor V and spectrin	[41,45,69]
SepA	*Shigella flexneri*	Intestinal inflammation, proteolytic activity toward synthetic peptides: Suc-Ala-Ala-Pro-Phe-pNA, Suc-Val-Pro-Phe-pNA and Suc-Phe-Leu-Phe-pNA	[70,71]
SigA	*Shigella flexneri*	Cytotoxin, Cleavage of casein, recombinant human α II spectrin Cell rounding and cell detachment	[72,73]
Boa	*Salmonella bongori*	Unknown	[74]
TagBC	UPEC, APEC	Autoaggregation, proteolytic effect on synthetic peptide: N-Benzoyl-L-arginine 4-nitroanilide cytopathic effect on human bladder cell lines	[31]

^a^ Bacteria known to produce these SPATEs. APEC, Avian pathogenic *E. coli*; DAEC, Diffuse Adhering *E. coli*; STEC, Shiga toxin-producing *E. coli*; UPEC, Uropathogenic *E. coli*; NMEC, Neonatal meningitis *E. coli*; EAEC, Enteroaggregative *E. coli*; EHEC, Enterohemorrhagic *E. coli*; EPEC, Enteropathogenic *E. coli*; ETEC, Enterotoxigenic *E. coli*.

**Table 2:** The potential H-NS binding sites on the promoter region of SPATEs as predicted by Virtual Footprint Software ^a^.

SPATEs	Potential H-NS Binding Sites
*boa*	^−97^GCAATAAACC^−88^ (−), ^−96^GCAATAAAAT^−87^ (−),^−80^GCTATAAAAA^−71^ (−)
*sigA*	^−179^TGGTTAGATA^−170^ (−),^−170^GTGATTGATT^−161^ (−), ^−19^CCGATATTTC^−10^ (−)
*pic*	^−159^CAGATAAAAC^−150^ (+), ^−109^TGCATTAATG^−100^ (−), ^−35^GGGATATAAA^−26^ (−)
*sepA*	^−176^ATGATAAAAA^−167^(+), ^−35^AAGATTAATT^−26^ (−)
*tsh/hbp*	^−164^CACATAAAGT^−155^ (−), ^−28^AAAATAAAAT^−19^ (−), ^−10^GTAATTAAAA^−1^ (+)
*espC*	^−300^ACCATTAAAA^−291^ (+), ^−299^CCATTAAAAT^−290^ (+), ^−111^GCCACAAACT^−102^ (−)
*espP*	^−280^TCGATTGTTA^−271^ (−), ^−96^CAGATAAATG^−87^ (−), ^−46^CTGATACATT^−37^ (+)
*pet*	^−177^ATGATTAATT^−168^ (+),^−42^AGGATTAAGA^−33^ (−),^−24^TCAATAAATG^−15^ (+)
*sat*	^−177^ACGATCAATT^−168^ (+),^−166^ACGATCAATT^−157^ (+),^−24^TCAATAAATG^−15^ (+)
*eatA*	^−88^GCTATCTATT^−79^ (+),^−71^ACAATAAATG^−62^ (+),^−40^TCCACACAAC^−31^ (−)
*eaaA*	^−314^ACCATACAGC^−305^ (−),^−124^GCGGTAAAAA^−115^ (−)
*tagB*	^−304^ACGAAAAAAA^−295^ (−),^−161^CTGATAAATA^−152^ (−),^−128^TCGATAAATG^−119^ (+)
*tagC*	^−256^GCAATTAATA^−247^ (+),^−62^TCGCTATATT^−53^ (+),^−56^ACTATAAATA^−47^ (−)
*sha*	^−187^CCCACAAATC^−178^ (−),^−48^TCCTTATATT^−39^ (+),^−32^TCAATAGATA^−23^ (−)
*vat*	^−296^TCCATATATC^−287^ (+),^−295^TGGATATATG^−286^ (−),^−107^GCTATATAAT^−98^ (−)

^a^ Virtual Footprint software [140] was used for in silico analysis of different SPATEs promoter region for putative regulatory H-NS binding sites and additional experimentation is required to confirm a role for H-NS in regulation of SPATE gene regulation. Pattern matching tool Virtual Footprint used specific position weight matrices (PWMs) to generate the top three potential binding sites upstream to the start codon with high scoring matches. (+/− strand).
